# Current Controversies in Antidepressant Therapy: A Patient-level Perspective

**DOI:** 10.1192/j.eurpsy.2023.45

**Published:** 2023-07-19

**Authors:** F. Hieronymus

**Affiliations:** ^1^Department of Clinicla Medicine, Aarhus University, Aarhus, Denmark; ^2^Institute of Neuroscience and Physiology, University of Gothenburg, Gothenburg, Sweden

## Abstract

**Abstract:**

The value of pharmacological antidepressants have been contested since they were first introduced in the 1960s, but the points of contention have varied over time. This session will examine and critically discuss some of the concerns that are commonly voiced today, with particular emphasis on the evaluation of efficacy. The session will cover topics such as the utility of dichotomized outcome measures (e.g., response and remission) and whether the use of these measures risk inflating apparent efficacy, whether antidepressant effect sizes are too small to be clinically meaningful, and whether there is individual variability in the response to pharmacological antidepressants.

**Disclosure of Interest:**

F. Hieronymus Speakers bureau of: I have received speaker’s fees from Janssen and H Lundbeck.

